# RETRACTION: Novel Circular RNA Hsa_circ_0036683 Suppresses Proliferation and Migration by Mediating the MiR‐4664‐3p/CDK2AP2 Axis in Non‐Small Cell Lung Cancer

**DOI:** 10.1111/1759-7714.70394

**Published:** 2026-10-09

## Abstract

**RETRACTION**: LiuR. , ZhangH. , XinJ. , XieS.‐Y. , JiaoF. , LiY.‐J. , ChuM.‐Y. , QiuJ. and YanY.‐F. , “Novel Circular RNA Hsa_circ_0036683 Suppresses Proliferation and Migration by Mediating the MiR‐4664‐3p/CDK2AP2 Axis in Non‐Small Cell Lung Cancer,” Thoracic Cancer 15, no. 27 (2024): 1929‐1945, 10.1111/1759-7714.15396.PMC1146293639113208

The above article, published online on 07 August 2024 in Wiley Online Library (https://onlinelibrary.wiley.com), has been retracted by agreement between the journal Editor‐in‐Chief, Tateaki Naito; and John Wiley & Sons Australia, Ltd. The retraction has been agreed upon following concerns raised by a third party. An investigation identified apparent splice sites around the circ‐36683 IgG band shown in Figure 7i. The authors cooperated with the investigation and provided supporting data; however, this did not resolve the concern, and doubts remain regarding the integrity of the figure. As a result, the editors have lost confidence in the reliability of the results and conclusions reported in this study. The authors disagree with the retraction.

**RETRACTION**: R. Liu , H. Zhang , J. Xin , S.‐Y. Xie , F. Jiao , Y.‐J. Li , M.‐Y. Chu , J. Qiu and Y.‐F. Yan , “Novel Circular RNA Hsa_circ_0036683 Suppresses Proliferation and Migration by Mediating the MiR‐4664‐3p/CDK2AP2 Axis in Non‐Small Cell Lung Cancer,” Thoracic Cancer 15, no. 27 (2024): 1929‐1945, 10.1111/1759-7714.15396.PMC1146293639113208

The above article, published online on 07 August 2024 in Wiley Online Library (https://onlinelibrary.wiley.com), has been retracted by agreement between the journal Editor‐in‐Chief, Tateaki Naito; and John Wiley & Sons Australia, Ltd. The retraction has been agreed upon following concerns raised by a third party. An investigation identified apparent splice sites around the circ‐36683 IgG band shown in Figure 7i. The authors cooperated with the investigation and provided supporting data; however, this did not resolve the concern, and doubts remain regarding the integrity of the figure. As a result, the editors have lost confidence in the reliability of the results and conclusions reported in this study. The authors disagree with the retraction.

